# Checkpoint inhibitors: Better outcomes among advanced cutaneous head and neck melanoma patients

**DOI:** 10.1371/journal.pone.0231038

**Published:** 2020-04-13

**Authors:** Nir Hirshoren, Roni Yoeli, Jonathan E. Cohen, Jeffrey M. Weinberger, Nadia Kaplan, Sharon Merims, Tamar Peretz, Michal Lotem

**Affiliations:** 1 Department of Otolaryngology / Head & Neck Surgery, Hadassah Hebrew-University Medical Center, Jerusalem, Israel; 2 Sharett Institute of Oncology, Hadassah Hebrew-University Medical Center, Jerusalem, Israel; 3 The Faculty of Medicine, The Wohl institute for Translational Medicine, Hadassah Medical Center, Hadassah Hebrew-University Medical Center, Hebrew University of Jerusalem, Jerusalem, Israel; 4 Radiology department, Hadassah Hebrew-University Medical Center, Jerusalem, Israel; Chang Gung Memorial Hospital at Linkou, TAIWAN

## Abstract

**Objective:**

The aim of this study was to investigate if the treatment outcomes of checkpoint inhibitors (CPI) in patients with advanced-stage skin head and neck melanoma (HNM) differs from outcomes in patients with non-HNM.

**Design:**

A retrospective cohort study of patients with unresectable AJCC stage III and stage IV, who received CPI between 2010 and 2017.

**Participants:**

Overall, 122 unresectable AJCC stage III and metastatic stage IV melanoma adult patients were treated with CPI during the study period (consecutive patients). The HNM group of patients was comparable with limbs and trunk melanoma group except different distant metastatic (M1a/b/c/d) pattern (p = 0.025).

**Main outcomes:**

Comparison of overall survival and clinical response to CPI in patients with advanced-stage skin melanoma of the head and neck with non-HNM.

**Results:**

We analyzed 38 patients with melanoma arising in the head and neck skin regions, 33 with melanoma of limbs and 51 with trunk melanoma. Most of the head and neck patients were men (89.5%), the average age of melanoma diagnosis was 61.4±16.7 years (range 16.4–85.6). More than a third of HNM group of patients (36.8%) were 70 years and older. Overall response rate (ORR) to CPI was 50% (CR 31.6% and PR 18.4%) in the head and neck study group of patients, compared to an ORR of 36.3% and 23.5% in melanoma of the limbs and of the trunk, respectively (p = 0.03). The median overall survival of HNM group of patients was 60.2±6.3 months, CI 95% [47.7–72.7], 63% were alive at 30 months, reaching a plateau. Whereas, the median survival time of limbs and trunk melanoma were 51.2 and 53.4 months, which did not reach significance.

**Conclusions and relevance:**

Response rate to CPI is significantly improved in patients with melanoma of the head and neck and they have a trend towards improved, long standing, overall survival.

## 1. Introduction

Cutaneous melanoma is more prevalent in the head and neck region relative to other body sites. Head and neck melanoma (HNM) is more likely to present with aggressive clinicopathological features with deeper Breslow thickness and advanced clinical stage [1]. However, a survival difference is still to be clarified [2].

The immune checkpoint inhibitors (CPI) treatment protocol for advanced-stage skin melanoma is not influenced by body region origin. Yet, our clinical impression argues for a better outcome among head and neck melanoma (HNM) patients. We hypothesize that "favorable" metastatic sites spread and known higher mutation burden (increased UV exposure) are associated with better CPI response.

Blocking antibodies of programmed death-1 (PD1) and cytotoxic T-lymphocyte–associated protein (CTLA-4) introduced a dramatic change in metastatic melanoma patients' treatment and prognosis, intensifying the proportion of responding melanoma patients[3]. The revolutionary concept behind these treatments is the reversibility of T lymphocytes dysfunctional state in the tumor environment endowing them regained anti-tumor activity[4]. Collectively called ‘checkpoint inhibitors’ (CPI), the two main classes targeting CTLA4 and the PD1/PD-L1 axis were broadly evaluated for their role in melanoma treatment, being the first disease to receive approval for non resectable metastatic disease[3]. Recently, the indications for CPIs in melanoma were broadened to include adjuvant treatment of patients with resectable AJCC (American Joint Committee on Cancer) stage III local-regional melanoma[5] and as neoadjuvant therapy and in stage IIB/C melanoma[6–9].

Checkpoint inhibitors tend to be more effective, demonstrating higher response rates, in patients whose tumors contain higher non-synonymous mutations (> 200 mutations per tumor, or 17 mutations per megabase) [10,11]. The significant association between head and neck melanoma and sunlight exposure has been demonstrated[12]. Etiologically, ultraviolet (UV) wavelength mediates faulty oxidized nucleotide base mispairing, causing mutagenesis[13,14] and chronic inflammation, which further enhances tumorigenesis[15–17].

Controversy still exists if there is a difference in BRAF mutation prevalence between HNM and non-HNM patients [18,19]. It should be noted that regardless of tumor skin site, advanced cutaneous melanoma with a BRAF mutation is eligible for treatment with BRAF inhibitors.

In accordance with our clinical impression, we demonstrate improved CPI response among head and neck melanoma patients as compared to other cutaneous melanoma sites.

## 2. Methods

### 2.1 Patients and study design

This study is a retrospective analysis of patients with advanced melanoma treated with CPI blocking CTLA-4 (Ipilimumab, BMS) or PD-1 (Nivolumab, BMS; pembrolizumab, Merck), in a tertiary medical center. The study was approved by the Hadassah Internal Review Board (HMO-0545-17) with a waiver for a written consent form. The data was extracted between the 1^st^ of January 2010 and 31^st^ of December 2017, with complete anonymization.

#### 2.1.1 Primary endpoints

Comparative analysis of clinical response to immune checkpoint inhibitors, overall and progression free survival (OS and PFS) in patients with unresectable AJCC stage III or metastatic stage IV cutaneous melanoma originating in the head and neck region, compared with melanoma originating in the skin of the trunk and limbs.

The medical records of all melanoma patients undergoing systemic therapy in the Sharett Institute of Oncology were surveyed. Surgical records at the Otolaryngology Department and pathological reports were reviewed. The following baseline parameters were retrieved from the time of initiation of CPI therapy: age, gender, Eastern Cooperative Oncology Group performance status (ECOG PS), M stage (AJCC 8^th^ edition), line of therapy for CPI agent, duration of therapy, lactate dehydrogenase (LDH) level, and BRAF mutation status. Efficacy included best response according to Immune-related Response Evaluation Criteria in Solid Tumors, (Ir-RECIST) version 1.1[20], PFS and OS. Non-evaluable patients were excluded from response analysis but were included in survival analyses.

#### 2.1.2 Patients

Included in this series are all patients with unresectable AJCC stage III or metastatic stage IV cutaneous melanoma treated with CPI at the Sharett Institute of Oncology- Hadassah Medical Center, between the 1^st^ of January 2010 and 31^st^ of December 2017.

To allow response evaluation, patients were required to have a minimal exposure to CPI, and therefore we excluded patients receiving ≤ 2 doses of CPI or with less than three months of follow up.

#### 2.1.3 Study and control groups

Patients were retrospectively divided into two groups: melanoma arising in skin of head and neck (group A) and melanoma arising in skin of limbs or trunk (group B). Uveal or mucosal melanoma are non-cutaneous and therefore omitted. Acral melanoma was excluded.

### 2.2 Adverse effects analysis

The CTCEA, version 5.0 [21] was used for adverse effects (AE) classification. Grade III CTCEA and above were considered as major AE. High dose steroid treatment for AE was defined as ≥40 mg prednisone (or its equivalent).

### 2.3. Statistical analysis

All statistical analyses were performed using IBM SPSS Statistics software version 24. Chi-square or Fisher's exact tests were used for comparison of qualitative and categorical parameters. The Student t-test and Mann-Whitney test were used for quantitative parameters and the ANOVA test for the comparison of more than two subgroups. Kaplan-Meier analysis was used for OS and PFS estimation. Cox regression model was used to test for association of different survival parameters. P-value of 0.05 or less was considered as statistically significant.

## 3. Results

### 3.1. Patients and disease characteristics

**Fig 1:**
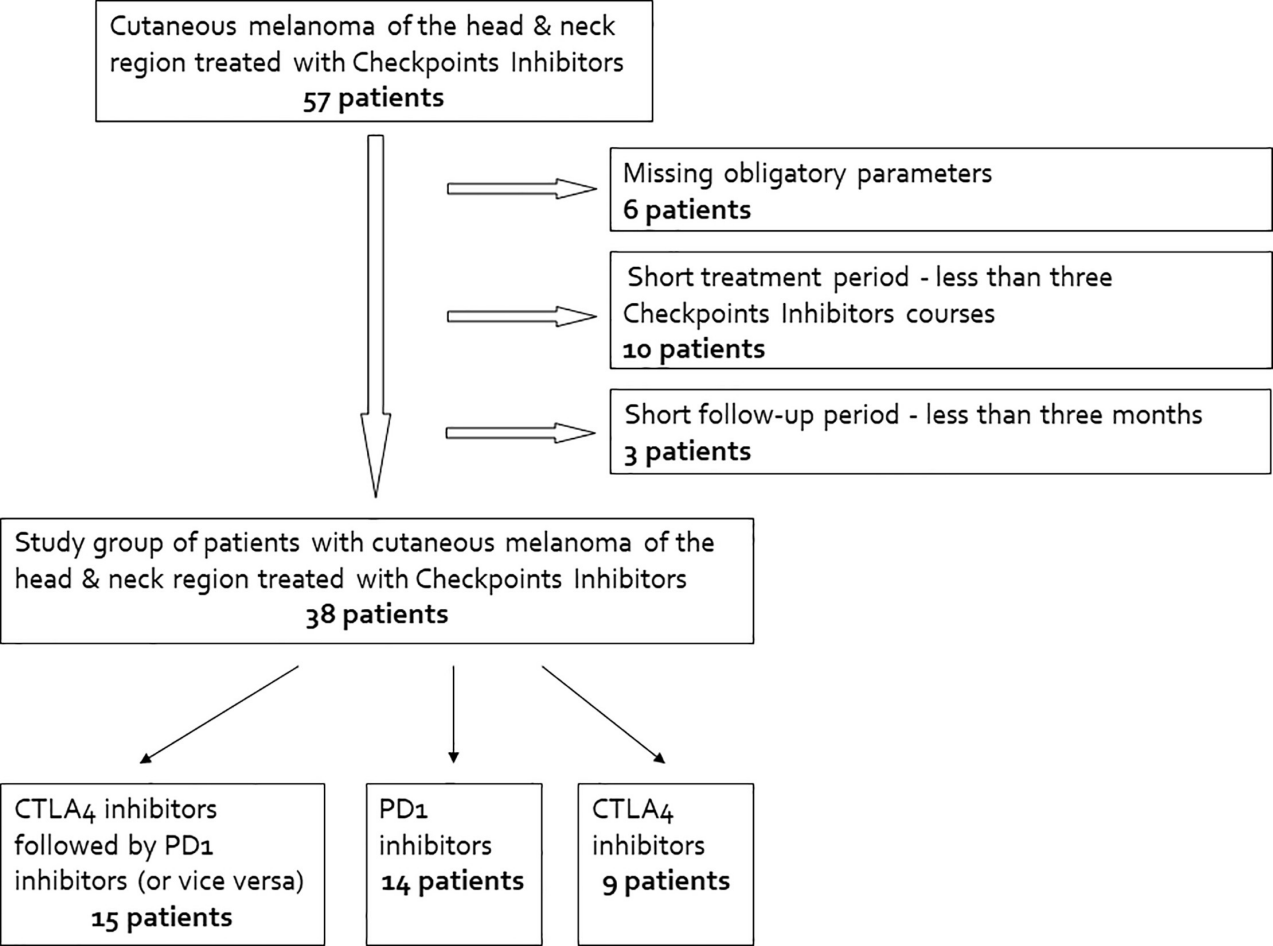
Study scheme.

Overall, 122 unresectable AJCC stage III and metastatic stage IV melanoma patients were treated with CPI during the study period. Of those, 38 were in the head and neck skin (Fig 1).

Group B, melanomas of limbs and trunk, included 33 and 51 patients respectively.

Most of the head and neck patients were men (89.5%, Table 1). The average age of melanoma diagnosis was 61.4±16.7 years (range 16.4–85.6). More than a third of Group A (36.8%) were 70 years and older compared to 21.4% in group B. Type II Fitzpatrick phototyping scale was recorded in 66% (all head and neck patients had either 1, 2 or 3 Fitzpatrick scale). More than half of the patients presented with advanced primary tumors; T3 in 18.8% and T4 in 34.4%. Furthermore, 44.8% had tumor ulceration and 75% had Clark level 4 or 5. The average number of mitoses was 6.5 per mm^2^ and mean tumor depth was 2.5 mm. Almost a third (31.6%) of the patients had prior cutaneous BCC or SCC before the melanoma. Eight patients had spindle, desmoplastic or neurotrophic melanoma. Six patients (15.7%) had elevated LDH levels (above the normal upper limit). Groups A and B were comparable for age (p = 0.09), proportion of elevated LDH levels (p = 0.56) and AJCC stage disease (p = 0.302) (Table 1).

**Table 1:** Comparison of demographic and main clinical data of the head and neck melanoma study group of patients (group A, n = 38) with limbs and trunk melanoma (group B, n = 84).

		Group A (Head and neck)	Group B (Trunk and Limbs)	P value
Average age at melanoma diagnosis (years)		61.4 ± 16.7	59 ± 13.5	0.09
Gender, N (%)	Female	4 (10.5%)	36 (42.9%)	<0.001
	Men	34 (89.5%)	48 (57.1%)	
Elevated LDH levels, N (%)		6 (15.7%)	10 (11.9%)	0.56
BRAF mutation prevalence, N (%)		9 / 29 (31%)	30 / 73 (41%)	P = 0.345
Disease AJCC* stage, N (%) (AJCC 8^th^ edition)	Stage III	7 (18.4%)	8 (9.5%)	0.302
	Stage IV	31 (81.6%)	76 (90.5%)	
Metastatic sub-categorization^†^, N (%)	M1a	1 (3.2%)	17 (22.4%)	0.025
	M1b	19 (61.3%)	19 (25%)	
	M1c	9 (29%)	25 (32.9%)	
	M1d	2 (6.5%)	15 (19.7%)	

*AJCC: American Joint Committee on Cancer

^†^ M1a: Distant metastasis to skin, soft tissue including muscle, and/or non-regional lymph node, M1b: Distant metastasis to lung, M1c: Distant metastasis non-CNS visceral sites and M1d: Distant metastasis to CNS

To note, there was a significant difference comparing distant metastatic (M1) sub-sites (M1a/b/c/d) among HNM and limbs/trunk melanoma groups of patients (p = 0.025). BRAF mutation V600 was found in 31% (9/29) of HNM compared to 41% (30/73) in non-HNM group of patients (p = 0.345). Not surprisingly, the majority of the HNM patients (N = 33) received 1^st^ line CPI treatment.

### 3.2. Treatment description

Six patients (15.8%) who presented with metastatic HNM, were treated by CPIs on diagnosis. Whereas, thirty-two patients received CPIs 26 months (median, range 0.5–162 months) after being diagnosed with HNM. Overall, the mean age for first course of CPI was 64.4±16 years (range 18.8–90.3). On CPI therapy initiation, seven of the patients (18.4%) had unresectable stage III and 31 patients (81.6%) had stage IV melanoma. Nine patients (23.7%) received CTLA4 inhibitor Ipilimumab, 14 patients (36.8%) had PD-1 inhibitor and 15 patients (39.5%) had CTLA4 and subsequently PD-1. No patient had a combined regimen. Median follow-up time was 27.4±19.1 months (range 3.6–61.4).

Most patients on CPI (81.5%) had adverse effects (AE). Skin AE were most common (52.6%) followed by gastrointestinal system (26.3%), fatigue and asthenia (23.6%), arthralgia and myalgia (21%), respiratory system (18.4%), fever (13.1%), endocrine system (10.5%), hepatobiliary system (10.5%) and weight loss (10.5%). Seven patients (18.4%) had major adverse effects; in four patients- CPI had to be stopped; in five patients-high dose steroids (≥ 40 mg prednisone) were administered. Of those 7 patients with major AE, two had target therapy prior CPI administration.

### 3.3. Efficacy analyses

**Fig 2:**
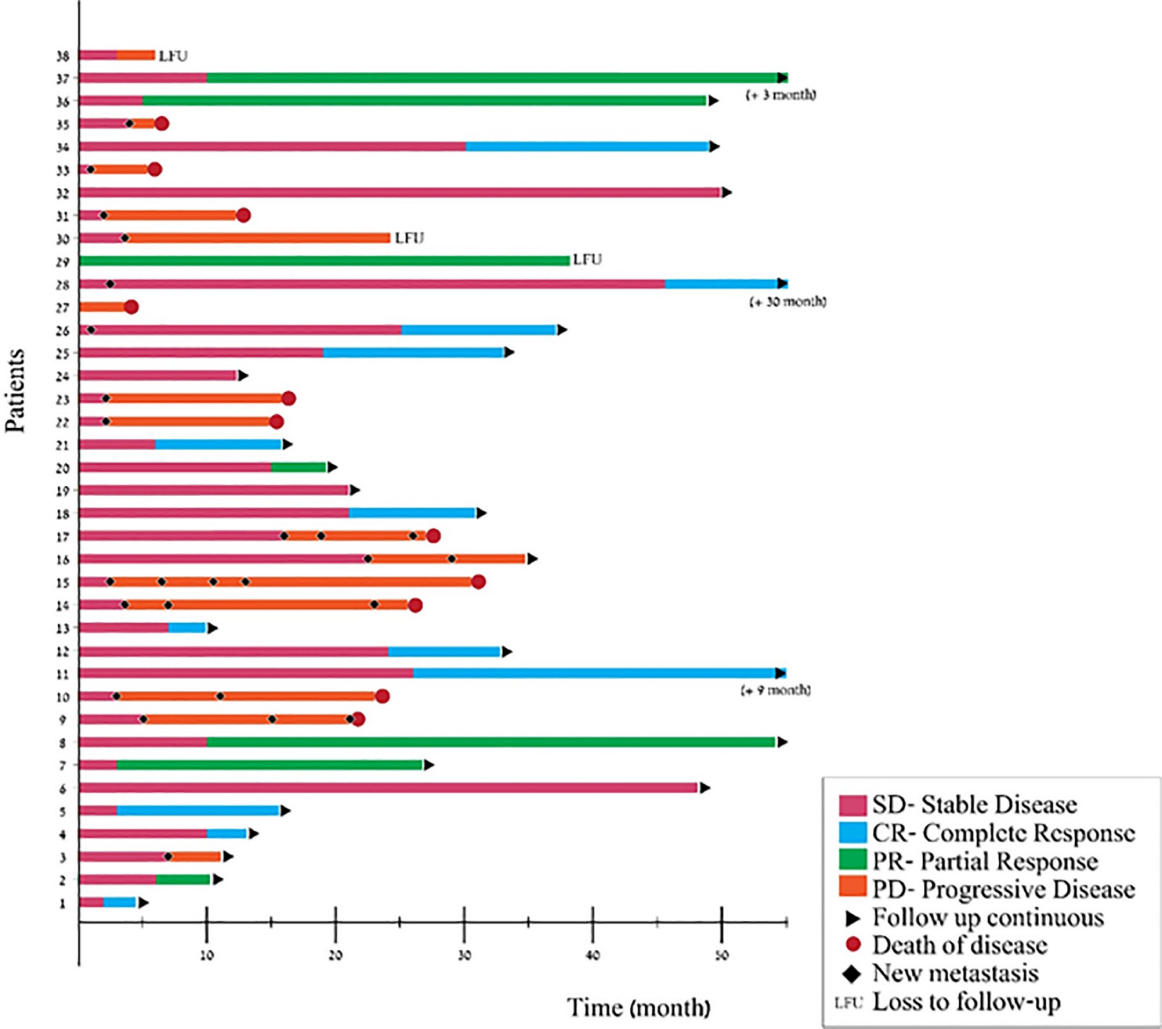
The individual response to checkpoint inhibitors (Swimmers' plot) among 38 patients with advanced head and neck melanoma patients.

The overall response rate in Group A, head and neck melanoma patients, was 50% (CR 31.6% and PR 18.4%). Stable disease was recorded in 10.5% of the patients and progressive disease in 39.5% (Fig 2).

**Fig 3:**
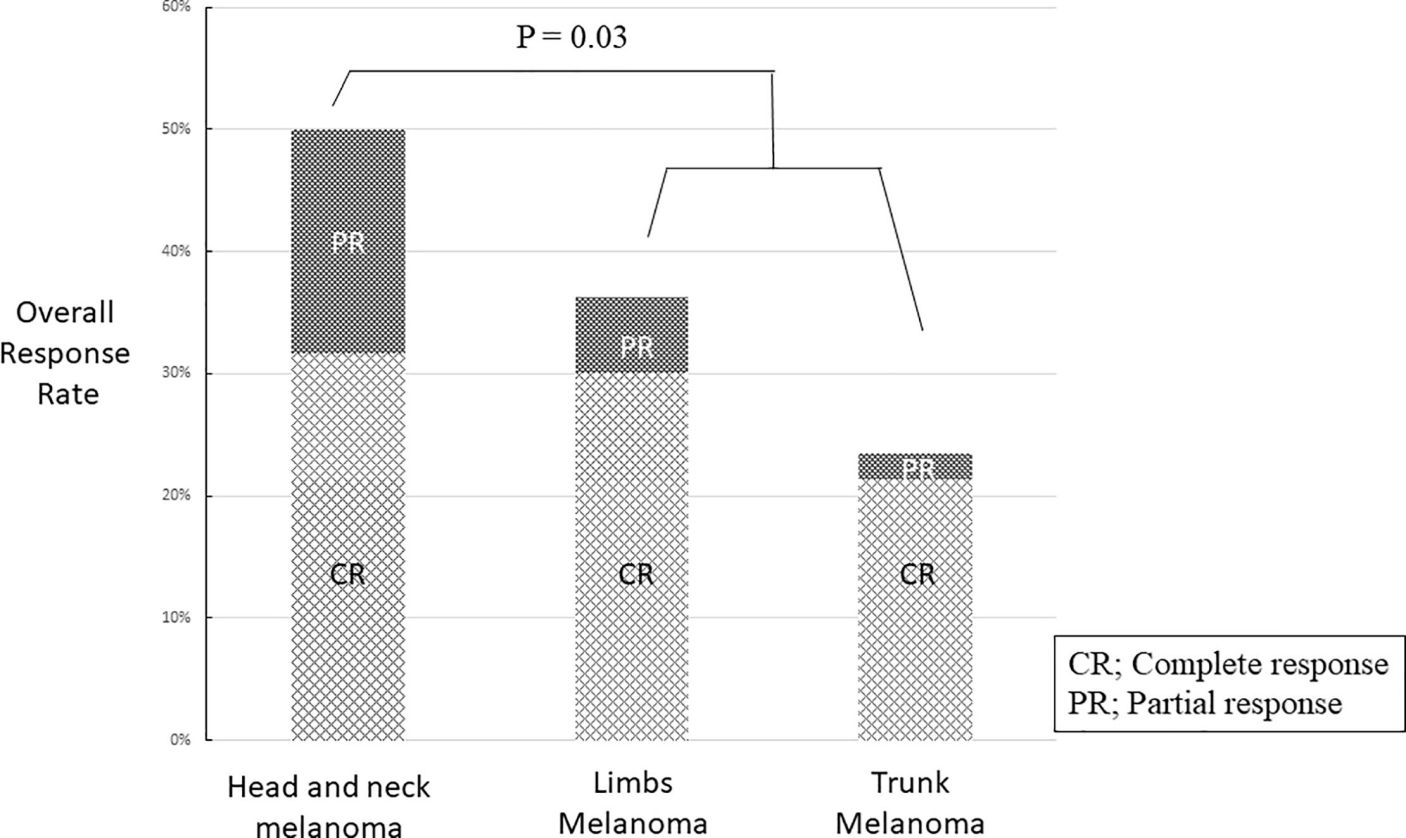
The overall response rate to checkpoint inhibitors of head and neck, limbs and trunk melanoma. Chi-Square test demonstrates a significant statistical difference (p value = 0.03).

In comparison, the ORR was 36.3% (CR 10 patients, PR 2 patients) and 23.5% (CR 11 patients, PR 1 patients) in limbs and trunk melanoma, respectively (Fig 3).

A significant ORR difference (p = 0.03) was found comparing head and neck with limbs and trunk melanoma.

For all patients, groups A and B (N = 122), we found a significant association between metastatic sub-categorization (M1a/b/c/d) and ORR (p<0.015). Further prominent association was found between CR and metastatic sub-categorization (p = 0.0077).

**Fig 4:**
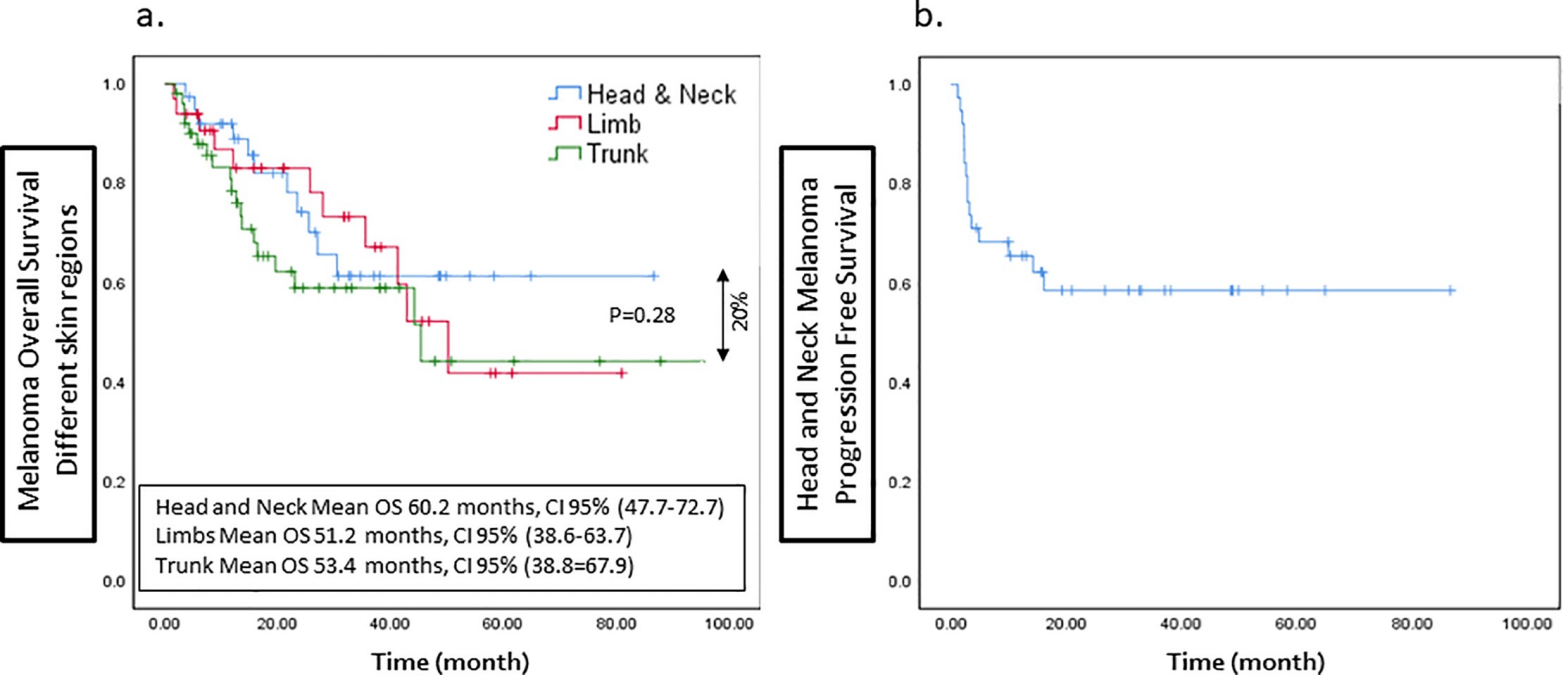
The overall survival of advanced head and neck (blue), trunk (green) and limbs (red) melanoma patients (A). The progression free survival of advanced Head and Neck melanoma patients treated with checkpoint inhibitors (B).

We found better survival data in patients with melanoma arising in the skin of the head and neck. Median overall survival (OS) of Group A patients was 60.2±6.3 months, CI 95% [47.7–72.7] and median progression free survival (PFS) was 52.8±6.7 months, CI 95% [39.5–66]. Patients in Group B had a median OS of 51.2±6.4 for melanoma arising in trunk and 53.4±7.4 for melanoma arising in limbs (Fig 4).

Sixty-three percent of the HNM patients were alive at 30 months, reaching a plateau, in contrast to continuing survival decline in Group B. A 20% better durable OS was demonstrated among the head and neck study group of patients, not reaching the statistical significance (p = 0.28).

A better OS (p = 0.03) was found in the elderly group of patients compared to the younger (less than 70 years old) age group (86% OS at 30 months follow up compare to 50% OS). There was no OS difference (p = 0.22) for those treated with Ipilimumab compared with PD-1 inhibitors treatment. In a multivariate analysis, no significant difference in OS was found for stage III compared to stage IV melanoma (p = 0.273), patients with major AE (p = 0.94), tumor thickness (p = 0.36), diverse head and neck skin sites (p = 0.135) or unique histology patterns (spindle, desmoplastic or neurotrophic melanoma) (p = 0.61). Primary tumor ulceration was an independent prognostic parameter, close to significance (p = 0.07) with a poor median OS of 27±2.2 months, CI 95% [2.5–31.4]. Primary tumor mitoses count was found to be a significant negative OS prognostic parameter (p = 0.0046, CI 95% [1.121–10.001]).

Distant metastases (M1) were detected in 31 patients before treatment administration and in 15 patients while treated with CPI. Altogether, lungs metastases (29 patients) were most common followed by unresectable neck lymph nodes (20), soft tissue (15), central nervous system (13), hepatic (11), adrenal (6), kidneys (5), spleen (4), thyroid (3), gastrointestinal (3), muscles (2), pancreas (1) and ovary (1). Non-resectable neck lymph nodes and lungs metastases had the best ORR (70% and 58.6%, respectively) while brain and bone metastases had the worst ORR (7% and 0%, respectively) (Table 2).

**Table 2:** Checkpoint inhibitor response rates in different metastatic sites among advanced head and neck melanoma patients.

Metastases Sites	Rate of Patients with site specific metastases	Overall Response Rate	Complete Response Rate	Progressive Disease Rate
Neck Lymph Nodes Number of patients (percentage)	20	14	13	4
	(52.6%)	(70%)	(65%)	(20%)
Lungs Number of patients (percentage)	29	17 (58.6%)	15 (51.7%)	7 (24.1%)
	(76.3%)			
Skin / Subcutaneous Number of patients (percentage)	15	8 (53.3%)	8 (53.3%)	6
	(39.5%)			(40%)
Distant Lymph Nodes Number of patients (percentage)	20	8 (40%)	7 (35%)	8 (40%)
	(52.6%)			
Hepatic Number of patients (percentage)	11	4 (36.3%)	3(27.3%)	6 (54.5%)
	(28.9%)			
Brain Number of patients (percentage)	13	1 (7%)	1 (7%)	10 (76.9%)
	(34.2%)			
Bone Number of patients (percentage)	4	0 (0%)	0 (0%)	3 (75%)
	(10.5%)			

In the 15 patients who progressed on CPI, the mean time to progression was 2.5 months (range 1–22) and 10 patients (66%) had new metastases within 12 weeks of CPI commencement. As expected, failure to respond to CPI was associated with worse prognosis, but even so, a median OS of 25.5 months was recorded.

## Discussion

In this retrospective patient cohort, we set to compare disease outcome of advanced melanoma arising in the skin of the head and neck region compared with other skin sites, with the emphasis on response to systemic immunotherapy with checkpoint inhibitors. We hypothesized that the apparent association between HN melanoma and sun exposure[22], higher mutation rate[23], together with a different distant spread pattern to a "favorable" sites leads to an improved immunotherapy response.

The study (head and neck melanoma) and control (limbs and trunk melanoma) groups did not differ in age, disease severity (all patients had unresectable AJCC stage III or stage IV) or proportion of patients with elevated LDH levels (Table 1). Yet, a favorable (p = 0.025) distant metastatic sub-categorization (M1a/b/c/d) was documented among the HNM patients. Better ORR (p = 0.015) was associated with distant metastatic sub-categorization pattern. Our series show that specific metastatic sites are associated with better CPI response including neck lymph nodes (70%) and lung spread (58.6%), compared with unfavorable metastatic sites such as brain (7%) and bones (0%). Similarly, metastatic sites and survival association following CPI treatment, was lately demonstrated among advanced melanoma[24], renal cell carcinoma and non-small cell lung carcinoma[25] patients.

Head and neck melanoma patients treated with CPI had a significantly better ORR (50%, p = 0.03) and an improved, durable, although non-significant, median overall survival of 60.2 months CI 95% [47.7–72.7]. Compared with the 63% of head and neck patients alive at 30 months, reaching a plateau, only 42% of the limbs and trunk melanoma group of patients survived. Altogether, the response and survival benefit of CPI among head and neck melanoma patients was better which could be partially attributed to favorable metastatic sites and hypothesized higher mutation rate.

Furthermore, better OS (p = 0.03) was demonstrated among the older group of patients (70 years and older) that comprised more than a third (36.8%) of the head and neck melanoma patients. This is in accordance with a recent report, describing a 50% response rate in melanoma patients older than 80 [26].

We have demonstrated a trend towards a negative association between overall survival and ulceration and tumor mitosis count supporting the role of primary tumor characteristics as determinants of overall prognosis. Unfavorable primary tumor characteristics were independently associated with poorer treatment response and survival. This approach, weighting the primary tumor parameters as predictors of response to treatment, is still controversial, albeit with some support from multicenter large studies[27,28].

Shortfalls of this study should be addressed. This was a retrospective analysis, and therefore is subject to the biases inherent to this study design. The number of patients with HNM treated with CPI is relatively low. We are therefore cautious in making further derivations of the study group into smaller subgroups (e.g. separate analysis of scalp melanoma and other head and neck skin regions, or separate comparison of the effect of PD1 versus CTLA4 inhibitors). The meticulous clinical and laboratory data collection may overcome its retrospective nature and the limited number of patients. A careful interpretation of the statistical analysis is mandatory and recommended in any future studies.

## Conclusions

This retrospective series shows that patients with melanoma arising in the head and neck have a significant better response to immune checkpoint inhibitors. Favorable metastatic distribution and hypothesized higher mutation rate could be among the reasons for our findings.

## Supporting information

S1 Data (XLSX)
